# Recurrence of chronic lymphatic leukemia as infiltration of the spinal cord

**DOI:** 10.25122/jml-2024-0321

**Published:** 2024-11

**Authors:** Josef Finsterer, Victor Rathkolb

**Affiliations:** 1 Neurology Neurophysiology Center, Vienna, Austria; 2 3rd Medical Department, Hanusch Krankenhaus, Vienna, Austria

**Keywords:** chronic lymphatic leukemia, CNS involvement, spinal cord infiltration, ataxia, pleocytosis

## Abstract

Central nervous system (CNS) involvement in chronic lymphocytic leukemia (CLL) is rare, and spinal cord infiltration as a presenting manifestation has only rarely been described. We present the case of a 65-year-old man with CLL, initially diagnosed at the age of 54, who had not received prior treatment. He presented with a six-week history of thoracic and epigastric pressure. Mild ataxia was found in the clinical exam. Clinical evaluation revealed leukocytosis (163 G/L; normal range: 4-10 G/L). MRI of the spine showed a mildly enhancing, T2 hyperintense central lesion extending between C3 and T8. There was a pleocytosis of 105 /micro/l consisting of neoplastic B-lymphocytes. The bone marrow biopsy diagnosed a relapse of CLL, and the patient was started on ibrutinib, which had a positive effect. This case highlights spinal cord infiltration as a rare initial manifestation of CLL relapse, presenting with non-specific symptoms such as thoracic and epigastric pressure and mild spinal ataxia.

## INTRODUCTION

Chronic lymphocytic leukemia (CLL) is a common B-cell leukemia in adults with an average onset between 60 and 70 years of age and a higher prevalence among men [1]. Patients are often asymptomatic and are diagnosed in the presence of lymphocytosis on routine blood tests [1]. Symptomatic patients usually present fatigue symptoms associated with splenomegaly or lymphadenopathy [1]. Infiltration of the central nervous system (CNS) or peripheral nervous system (PNS) by neoplastic lymphocytes is rare and includes meningeosis, hemorrhage, confusion [2], optic neuropathy, pituitary dysfunction [3], cerebellar dysfunction, spinal cord compression [4], and cranial nerve dysfunction [5]. Infiltration of the spinal cord by neoplastic B-lymphocytes has rarely been described as a complication of CLL [6-8]. Here, we report a previously untreated patient with CLL in whom the relapse of CLL manifested with infiltration of the spinal cord 11 years after the initial diagnosis.

## CASE REPORT

The patient is a 65-year-old man with CLL, first diagnosed at the age of 54 with lymphocytosis and lymphadenopathy. The biopsy revealed that the lymph nodes were enlarged by the proliferation of small proliferating lymphocytes in an ill-defined nodular pattern. A bone marrow biopsy and flow cytometry revealed B-cell proliferation positive for CD5 and CD23 and the kappa light chain restriction. As the lymphocytosis regressed spontaneously, a wait-and-see attitude was adopted, and the patient did not receive any treatment since this initial CLL diagnosis. His medical history included chronic migraine without aura with a low frequency of attacks since adolescence, transient facial paralysis of unknown cause at the age of 18, and gastritis and reflux esophagitis since the age of 52. At the age of 55, he suffered a complete paralysis of the oculomotor nerve on the right side, which completely regressed, but the etiology remained unclear as cerebrospinal fluid examinations and imaging studies remained normal. At the age of 64 years, 16 months before the present admission, he suffered a herpes zoster virus (HZV) infection in the right lower abdomen in dermatome Th12, followed by post-zoster neuralgia in a similar distribution.

At the age of 65, he was admitted to the hospital because he had been complaining for 6 weeks of tiredness, fatigue, and exhaustion, as well as a stabbing and throbbing sensation in the sternum, a constant feeling of pressure in the lower thorax and epigastrium that was not related to food intake, vibrations in the abdominal muscles, listlessness and a lack of motivation for physical activity. His last medication included pantoprazole, pregabalin (150 mg/d), and vitamin D. The clinical neurological examination revealed myopia, anisocoria (right < left), positive pyramidal signs on the right upper limb, postural tremor (left > right), mild ataxia on the lower limbs (left > right), ataxic gait, disturbed line walk and a tendency to fall when walking blind.

**Figure 1:**
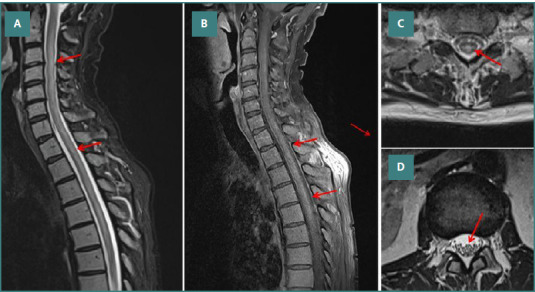
MRI of the spinal cord showing an (A, B) elongated, T2-hyperintense lesion C3 to T8 which mildly enhanced after application of contrast medium. C, D, on transverse sections the lesion was located centrally around the central canal

The ECG showed a sinus rhythm. Blood examination revealed a slight elevation of C-reactive protein (CRP), glutamate oxalate transaminase (GOT), glutamate pyruvate transaminase (GPT), and lactate dehydrogenase (LDH), marked leukocytosis and mild anemia (Table 1). The viral panel in serum, including HZV, HIV, and SARS-CoV-2, was negative. Antibodies against Borrelia, Lues, and ANCA were negative. Magnetic resonance imaging (MRI) of the spine showed a centrally located, elongated, T2 hyperintense lesion between C3 and T8, which increased only slightly after contrast administration (Figure 1A-D). Cerebral MRI showed nonspecific gliotic patches that were not suggestive of multiple sclerosis, neuromyelitis optica spectrum disorder (NMOSD), or myelin oligodendrocyte glycoprotein-associated disease (MOGAD). Antibodies against MOG, AQP4 and NMO were negative. CSF examination showed a mild pleocytosis of 105/microl (n, 0-4/microl) and increased CSF proteins due to blood-brain barrier (BBB) disruption. The oligoclonal bands (OCBs) were negative. The viral panel was negative. The antineuronal antibodies were negative. CSF cytology revealed neoplastic lymphocytes. Visually evoked potentials (VEPs) were normal. Ultrasound examination of the abdomen revealed only mild splenomegaly.

**Table 1:** Laboratory parameters during hospitalization

Parameter	RL	hd5	hd6	hd16	hd19
Leucocytes	4-10G7l	163.2	118.1	139.0	120.9
Lymphocytes	0.8-3.5G/l	nd	nd	44.3	nd
Erythrocytes	4.2-5.5T/l	3.9	3.8	3.8	3.6
Hemoglobin	14-18g/dl	11.8	11.8	10.8	11.0
Hematocrit	42-51%	36.8	36.7	34.8	34.8
Thrombocytes	150-400G/l	140	131	120.8	134
GOT	0-49U/l	nd	nd	73	nd
GPT	0-49U/l	58	nd	51	nd
CRP	<5mg/L	29.4	26.7	3.9	nd

CRP, C-reactive protein; GOT, glutamate-oxalate transaminase; GPT, glutamate pyruvate transaminase; hd, hospital day; nd, not done; RL, reference limits

Bone marrow biopsy and flow cytometry showed a proliferation of B-lymphocytes (93% CD19+ B lymphocytes and 39% CD20+ B-lymphocytes) with kappa light chain restriction positive for CD5+ and CD23+, but there was also a second population of CD5+ B-lymphocytes with lambda light chain restriction. Chemotherapy with the tyrosine-kinase inhibitor ibrutinib was prescribed. This treatment was well tolerated and subsequently led to a slow regression of the initial symptoms until discharge.

## DISCUSSION

The patient is of interest because of the infiltration of the spinal cord by malignant B lymphocytes as CNS involvement in CLL. To date, spinal cord involvement in CLL has been rarely reported [6-9]. Spinal cord involvement in these patients manifested as quadriparesis [6], NMOSD [7], autoimmune myelitis [8], or neck pain and urinary retention [9]. In addition to direct infiltration of the CNS, secondary autoimmune complications of CLL can also occur in the CNS or PNS, such as progressive multifocal leukoencephalopathy (PML), NMOSD, paraneoplastic disease, neuromuscular disease or peripheral neuropathy [9], all of which were absent in the index patient. In the index patient, there was no evidence of cerebral involvement in CLL, as previously reported [10].

In general, CNS infiltration in CLL is rare and tends to involve the brain but not the spinal cord [11]. CNS involvement in CLL can manifest as progressive cognitive impairment, headaches, balance problems, cranial nerve lesions, and weakness [12,13]. Optic perineuritis has also been reported in some cases [14]. Seizures or hemiparesis may also occur in cases with parenchymal mass lesions [15]. However, CNS involvement can also be asymptomatic, making diagnosis difficult [16]. Treatment options for CNS involvement in CLL include ibrutinib, bendamustine, rituximab, and venetoclax [11]. If patients develop resistance to any of these treatments, CNS involvement can be controlled with high-dose methotrexate in combination with pomalidomide [11].

In the case of the index patient, the combination of tiredness, fatigue, exhaustion, pronounced leukocytosis, repeatedly elevated CRP, and slightly elevated GOT, GPT, and lactate dehydrogenase (LDH), along with findings of increased lymphocytes in the bone biopsy and splenomegaly, pointed toward the relapse of CLL. Recurrence of the HZV infection was ruled out as the PCR for HZV in the CSF was negative, and there were no corresponding skin lesions. BBB dysfunction was present, and mild pleocytosis of malignant lymphocytes was noted. Furthermore, MOGAD, NMOSD, and vasculitis were excluded. Evidence against a demyelinating disease included negative antibodies, normal VEPs, cerebral MRI findings with no typical demyelinating lesions, negative OCBs, and the absence of autochthonous production of immunoglobulins. Elevated CSF proteins were attributed to BBB dysfunction. Additionally, the localization, size, and staining behavior of the observed lesions did not indicate a demyelinating process.

## CONCLUSION

In summary, this case shows that spinal cord infiltration can be the initial manifestation of CLL relapse and that it can manifest with thoracic and epigastric pressure and mild spinal ataxia only. In patients with gastritis and gastroesophageal reflux, the sensation of pressure in the same distribution as gastritis must be taken seriously, and a thorough diagnostic workup must be performed in order to overlook a CLL recurrence.

## Data Availability

Data that support the findings of the study are available from the corresponding author.
